# Effects of different grassland utilization methods on the germinable soil seed bank of the Hulunbuir meadow steppe

**DOI:** 10.3389/fpls.2023.1230725

**Published:** 2023-10-03

**Authors:** Ruirui Yan, Tianqi Yu, Hongmei Liu, Shijie Lv, Baorui Chen, Yanling Wu, Guoping Que, Zhijun Wei, Lijun Xu, Xiaoyu Zhu, Guixia Yang, Xiaoping Xin

**Affiliations:** ^1^ Hulunber Grassland Ecosystem National Observation and Research Station/State Key Laboratory of Efficient Utilization of Arid and Semi-arid Arable Land in Northern China, the Institute of Agricultural Resources and Regional Planning, Chinese Academy of Agricultural Sciences, Beijing, China; ^2^ Grassland Institute of Inner Mongolia Academy of Forestry Sciences, Inner Mongolia, Hohhot, China; ^3^ College of Science, Inner Mongolia Agricultural University, Inner Mongolia, Hohhot, China; ^4^ College of Life Science and Technology, Inner Mongolia Normal University, Inner Mongolia, Hohhot, China; ^5^ Inner Mongolia Taiwei Ecological Technology Co., Ltd., Hohhot, China; ^6^ Agro-Environmental Protection Institute, Ministry of Agriculture and Rural Affairs, Tianjin, China

**Keywords:** hulunbuir grassland, grassland utilization, soil seed bank, soil germination seed bank, perennial grass, upper growth grasses

## Abstract

Seed banks are crucial regenerative resources for aboveground vegetation. The pattern of their changes holds immense significance in understanding alterations in the belowground seed bank. This understanding is pivotal for uncovering both short-term and long-term shifts in plant communities. Additionally, it contributes to the restoration of grassland ecosystems. To better protect grassland biodiversity and provide a theoretical basis for the restoration of degraded grasslands, in this study, the germination characteristics of soil seed banks in free-grazed, enclosed and mown areas were compared, and the results were combined with those of previous studies for a comprehensive analysis. The density of soil seed bank and perennial forage soil seed bank were significantly affected by different grassland utilization and soil depths. Grazing and enclosure grassland utilization methods increased the content of the soil seed bank, and mowing reduced the content of the seed bank. The soil seed bank density of perennial grasses accounted for the highest proportion under grazing, followed by mowing, and its lowest proportion was observed in the enclosures. Grazing not only facilitated the germination of the perennial grass seed bank but also substantially augmented its content. Mowing inhibited the germination of the upper growth grasses seed bank, which was particularly significant in the 0-2 cm soil layer under grazing. The content of the upper growth grasses seed bank affected the total seed bank to a certain extent, mainly in the 5-10 cm layer. The general correlations among the perennial grasses, upper growth grasses and soil germination seed bank resulted in 84.58% information extraction, and this information has practical significance for grassland ecological restoration.

## 1 Introduction

Soil seed banks are important regenerative resources for aboveground vegetation because they offset fluctuations in seed production and quality when plant populations are disturbed (Dullinger and Hulber, 2011). They constitute one (Resilience) of the 3 dimensions (Robust or time-stability, Resistance、Resilienc) of ecosystem stability (Godfree et al., 2011). Thus, the variability of the belowground seed bank profoundly affects the diversity of aboveground plants in grasslands (Chesson and Warner, 1981). Understanding changes in the belowground seed bank is of great significance for revealing long-term and short-term changes in plant communities and for the restoration of grassland ecosystems (Thompson, 1989; Tóth et al., 2022).

Grassland use patterns can affect changes in the soil seed bank through many ways. Based on the existing research on the seed bank of grasslands according to different use patterns, we found that the impact of different grassland use patterns on grassland ecosystems is complex and diverse, which leads to differences in the changes in grassland soil seed banks under different utilization patterns, and the mechanisms that cause such differences are different and result from a combination of causes. Grassland use patterns can affect all stages of forage growth, especially the reproductive stage, resulting in changes in the plant seed bank. For example, grazed and mown grasslands are affected by livestock grazing and machine mowing; forage grasses on these grasslands are often unable to reach their reproductive growth stage, and even when they reach the reproductive growth stage, the plant seeds are fed or mowed in the later stage, thereby changing the size of the seed bank (Hartnett, 2002). Different grassland utilization methods can also change the size of the seed bank by changing the soil texture and environment (Walters, 1998). For example, grazing can change the soil texture, water content and nitrogen content of the soil through livestock trampling and manure deposition and further change the germination rate of soil seeds, seed setting rate of forage grasses (Gao et al., 2020) and decay rate of soil seeds (Papendick, 1972). Grassland utilization methods can also further change the seed longevity and seed activity of the soil seed bank by altering the microenvironment of the soil surface. For example, the presence of litter changes the temperature and humidity of the soil surface, which in turn changes the activity of soil seeds. To a certain extent, litter indirectly increases the density of the surface soils of grasslands by increasing the volume of the soil and ultimately reduces the density of the seed bank in the soil (Sayer, 2006; Zhang et al., 2017).

There is a direct link between a grassland germinable soil seed bank and its burial depths. In general, the deeper the seeds are buried, the longer the seeds survive, so the burial depths of the soil seed bank often indirectly indicates the longevity of the soil soil seed bank (Erfanzadeh et al., 2017; Erfanzadeh et al., 2020). Seeds in a soil seed bank are less likely to reach the surface as the depths of burial increases. soil seed banks at different depths will have different seed contents and different changes in seed germination rates due to their soil environment. The temperature, humidity, light and other conditions of soils at different depths will change the physiological processes of seed dormancy, activity, longevity and germination, thereby changing the soil seed bank (Kasperbauer and Hunt, 1988; Baskin and Baskin, 2014). The size of the soil seed banks is also influenced by the type of aboveground plants that are present. Perennial forages have a relatively positive response to competition arising from resource limitations and have different reproductive strategies in different environments, while the differences in the autotrophic plants of the upper growth grasses and lower flourishing grasses have different effects on the soil seed bank under different utilization methods (Diaz et al., 2004).

Grazing, fencing-in and mowing are the main types of grassland utilization patterns in northern China (Wang et al, 2020). It is of great significance to study the effect of different grassland utilization methods on the soil seed bank of grasslands to understand grassland biodiversity and guide grassland ecosystem restoration. For this reason, we carried out the following research. The main purpose was to address the following two questions: (1) How does the distribution of the soil seed bank at different grassland depths change under the three utilization methods? (2) How does the soil seed bank content of upper growth grasses and perennial grasses affect the overall soil seed bank content? The answers to these questions can provide empirical support for the ecological restoration of grasslands.

## 2 Materials and methods

### 2.1 Overview of the experimental site

This study was conducted in the long-term observation sample sites of the *Leymus chinensis* meadow grasslands at the Hulunbuir Grassland Ecosystem Experiment Station of the Chinese Academy of Agricultural Sciences. The study sites were located in production brigade 6, 11 and 12 of the Xeltala cattle breeding farm in Hulunbuir, Inner Mongolia. The basic conditions of the different sample sites utilized in each production brigade were as follows (**Table 1**).

**Table 1:** Basic conditions of different breeding farms.

Indicators	Location Name		
	Production brigade 6	Production brigade 11	Production brigade 12
Longitude	E120°2′47″-3′36″	E120°6′50″-7′28″	E120°2′47″-3′36″
Latitude	N49°19′32″-19′51″	N49°20′52″-21′16″	N49°19′32″-19′51″
Average annual temperature	-2.2 ℃	-2.4 ℃	-2.2 ℃
Annual precipitation	351 mm	350 mm	351 mm
Soil type	Dark chestnut calcium soil	Dark chestnut calcium soil	Dark chestnut calcium soil
Major populations	*Leymus chinensis, Stipa baicalensis, Artemisia tanacetifolia, Pulsatilla turczaninovii*, *Cleistogenes squarrosa*	*Leymus chinensis, Stipa baicalensis, Artemisia tanacetifolia, Pulsatilla turczaninovii*, *Cleistogenes squarrosa*	*Leymus chinensis, Stipa baicalensis, Artemisia tanacetifolia, Pulsatilla turczaninovii*, *Cleistogenes squarrosa*
Community-building species	*Leymus chinensis*	*Leymus chinensis*	*Leymus chinensis*
Grazing treatment	Fencing, pasture-mowing, free grazing	Fencing, pasture-mowing, free grazing	Fencing, pasture-mowing, free grazing

### 2.2 Soil seed bank sampling method

Three sample plots (each with an area of 15 m×15 m) were randomly selected under each utilization method of production brigade 6, 11 and 12 of the cattle breeding farm, and three 1 m×1 m quadrats were randomly selected within each sample plot. Six samples were taken from points in each sample square using a soil ring knife with a diameter of 5 cm, and the samples were taken at depths of 0~2 cm, 2~5 cm and 5~10 cm; then, the soils obtained from the same sample square of the same soil layer were mixed. Therefore, the number of samples was 3 production team × 3 utilization methods × 3 sample plots × 3 sample squares × 3 layers of soil samples = 243.

### 2.3 Soil seed bank germination tests

The seed bank was collected in August 2018, after which the air-dried soil was placed in a pergola (without heating equipment). The seeds began to germinate from August to October 2019, and the seeds that could germinate entered the state of germinable seeds after undergoing vernalization in winter and spring. Germination trays (17 cm in diameter and 3 cm in height) were selected, and each soil sample was placed in a germination tray at a thickness of 1-2 cm with a substrate of vermiculite, and germination was carried out for 45 d (45d germination is obtained according to the germination rate accumulation curve, when the number of germinable seeds is almost no longer increased, the state is delayed for another week, to ensure that the number of seeds that can germinate almost all germination.). Water was sprayed from time to time during this period to ensure that the soil samples were moist. During the germination process, seedlings were identified by species identification. Seedling species identification was generally performed based on the seedling morphological characteristics combined with seedling colour, seedling odour and seed germination characteristics. For species that could be identified, the number of germinations was recorded daily, and for species that could not be identified, the number of germinations was recorded daily, and the seedlings were transplanted for later species identification.

### 2.4 Statistical analysis

Data testing: The Univariate procedure of the SAS software was employed with the NORMAL keyword to perform tests for normality on the viable seed count of soil, viable seed count of perennial grasses, and viable seed count of upper growth grasses. In cases where the data did not conform to a normal distribution, square root or logarithmic transformation was applied.

ANOVA: The data for the number of soil germinable seeds, the number of perennial grass germinable seeds, and the number of germinable seeds of upper growth grasses were subjected to ANOVA. First, two-factor ANOVA was performed for soil layers (0-2 cm, 2-5 cm and 5-10 cm) and utilization methods (fenced-in area, pasture and free range). Then, one-way ANOVA was conducted for different utilization methods for the same soil layer and different soil layers of the same utilization method. Finally, Duncan’s multiple comparison tests were conducted for indicators with significant ANOVA results (in one-way ANOVA). The homogeneity test of Levene variance was also performed. SAS software was used for ANOVA. The results of the pairwise analysis were plotted as bar graphs in Sigmaplot 14.0, and the error line (SE) was marked, while the results of multiple comparisons were characterized using the letter marking method (Note: the same letter means no significant difference between comparisons, P>0.05).

General correlation analysis: General correlation analysis was conducted for the germinable soil seed bank of perennial grasses (PG), upper growth grasses (UG), and all grasses (TG), where the number of germinable seeds corresponding to the 0-2 cm, 2-5 cm, and 5-10 cm soil layers were the primary variables within the general variables, and the germinable soil seed bank of perennial grasses and upper growth grasses; the soil germinable soil seed bank of perennial grasses and topsoil grasses; and the germinable soil seed bank of the soil were typical variables. The typical variables of the soil germinable soil seed bank were obtained by first analysing the correspondence between perennial grasses and the soil germinable soil seed bank, and then a typical correlation analysis was performed between the typical variables of the germinable soil seed bank of the upper growth grasses and the soil germinable soil seed bank. Finally, the typical variables of the germinable soil seed bank of perennial grasses, upper growth grasses, and the total soil were analysed by simple correlation analysis. The CANCORR procedure was carried out for SAS for the typical correlation analysis, and the CORR procedure was used for SAS for the simple correlation analysis.

## 3 Results

### 3.1 Species occurrence in different grassland utilization methods

Overall, under the three utilization methods, *Astragalus melilotoides*, *Artemisia tanacetifolia*, and *Thalictrum squarrosum* all germinated at different depths, while the seeds of other plant species did not germinate at different depths; *Leymus chinensis* did not germinate in 5-10 cm of soil in the fenced grassland of the production brigade 6 but germinated at different soil depths in other grasslands; *Pulsatilla turczaninovii*, *Cleistogenes squarrosa*, *Adenophora stenanthina* and *Stipa baicalensis* mainly germinated in 0-5 cm depths soil, and only a small number germinated in 5-10 cm soil. The detailed germination data for plant seeds is shown in **Table 2**.

**Table 2:** Occurrence of soil seed bank species under various grassland use practices on different farms.

Plant populations	Top grass	Perennial Grasses	Soil layer	Brigade 6			Brigade 11			Brigade 12		
				CK	CP	FG	CK	CP	FG	CK	CP	FG
*Leymus chinensis*	yes	yes	S1	+	+	+	+	+	+	+	+	+
			S2	+	+	+	+	+	+	+	+	+
			S3	—	+	+	+	+	+	+	+	+
*Astragalus melilotoides*	yes	no	S1	+	+	+	+	+	+	+	+	+
			S2	+	+	+	+	+	+	+	+	+
			S3	+	+	+	+	+	+	+	+	+
*Artemisia tanacetifolia*	yes	no	S1	+	+	+	+	+	+	+	+	+
			S2	+	+	+	+	+	+	+	+	+
			S3	+	+	+	+	+	+	+	+	+
*Pulsatilla turczaninovii*	no	no	S1	+	+	+	+	+	+	—	+	+
			S2	+	+	—	+	+	+	+	+	+
			S3	—	—	—	—	—	—	—	—	—
*Allium tenuissimum*	no	no	S1	+	+	+	+	+	+	+	+	+
			S2	+	+	—	+	+	+	+	+	+
			S3	+	—	+	+	+	+	+	+	—
*Cleistogenes squarrosa*	no	yes	S1	+	+	+	+	+	+	+	+	+
			S2	+	+	+	+	+	+	+	+	+
			S3	—	—	+	+	—	+	—	—	—
*Thalictrum squarrosum Steph*	yes	no	S1	+	+	+	+	+	+	+	+	+
			S2	+	+	+	+	+	+	+	+	+
			S3	+	+	+	+	+	+	+	+	+
*Adenophora stenanthina*	yes	no	S1	+	+	+	+	+	+	+	+	+
			S2	+	—	+	+	+	+	+	+	—
			S3	+	—	—	—	—	—	—	—	+
Stipa baicalensis	no	yes	S1	+	+	+	+	+	+	+	+	+
			S2	+	—	+	+	+	+	+	+	+
			S3	+	—	—	—	—	—	+	—	+
Others	no	no	S1	+	+	+	+	+	+	+	+	+
			S2	+	+	+	+	+	+	+	—	+
			S3	+	+	—	+	+	+	+	+	+

### 3.2 Effects of different grassland utilization methods on the soil germinable soil seed bank

In the ANOVA based on the effect of utilization method and soil depths on the germinable soil seed bank (**Table 3**), it was found that both utilization methods and soil depths had highly significant effects on the germinability of soil seeds (P<0.01), whereas the interaction between utilization method and soil depths did not have a significant effect on seed germinability (P=0.8792).

**Table 3:** Analysis of variance (ANOVA) for the effect of utilization method and soil depths on the soil germinable seed bank.

Source	DF	SS	MS	F Value	Pr>F	R-Square
Model	8	72.98	9.12	41.66	<.0001	0.8224
Utilization mode	2	2.75	1.38	6.29	0.0030	
Soil layer	2	69.96	34.98	159.77	<.0001	
Utilization mode × Soillayer	4	0.26	0.06	0.30	0.8792	
Error	72	15.76	0.22			
Total	80	88.74				

**Figure 1:**
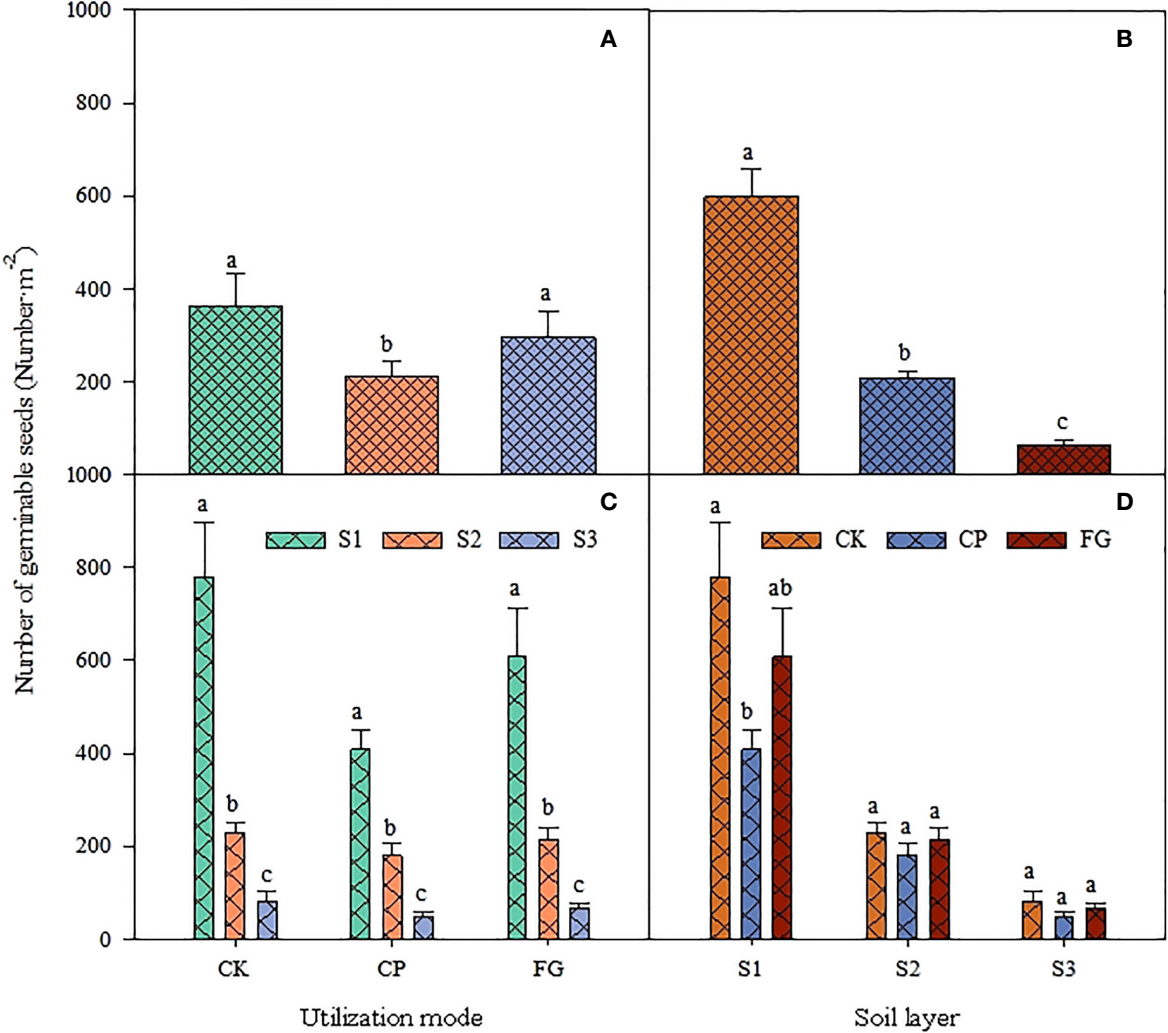
Response of soil germinable seed number to utilization method and soil depth. CK, CP and FG represent the fenced area, pasture-mowing and free grazing area, respectively; S1-S3 represent the 0-2cm, 2-5cm and 5-10cm soil layer, respectively. There is no significant difference between the same lowercase letters in the figure, P>0.05

For grassland utilization (**Figure 1-A**), the number of germinable seeds in the subsurface soil seed bank was CK (fenced area) > FG (free-grazing area) > CP (mowing area), where the number of germinable seeds in the soil seed bank in the grassland area was significantly lower than that in the enclosed and free-grazing areas (P<0.05, **Figure 1-A**), while the number of germinable seeds in the enclosed area was slightly higher than that in the free-grazing area, but there was no significant difference (P>0.05, **Figure 1-A**); within each grassland use, the number of germinable seeds was significantly lower in the order S1 (0-2 cm) > S2 (2-5 cm) > S3 (5-10 cm) (**Figure 1-C**). The differences in the number of seed germinations under each grassland utilization method showed the same characteristics at each soil depths: CK > FG > CP. The number of seed germinations in the enclosed area was significantly higher than that in the mown area in soil layer S1 (0-2 cm), but in soil layers S2 (2-5 cm) and S3 (5-10 cm), the differences resulting from the three utilization methods were not significant (**Figure 1-D**), and the distribution for germinable seed number in the different soil layers all showed the trend S1 (0-2 cm) > S2 (2-5 cm) > S3 (5-10 cm), and the difference was significant (**Figure 1-B**). Compared with the enclosed grassland, mowing inhibited the germination of the soil seed bank in the soil, especially in the 0-2 cm soil layer.

### 3.3 Effects of different grassland utilization methods on the soil seed bank of perennial grasses

**Figure 2:**
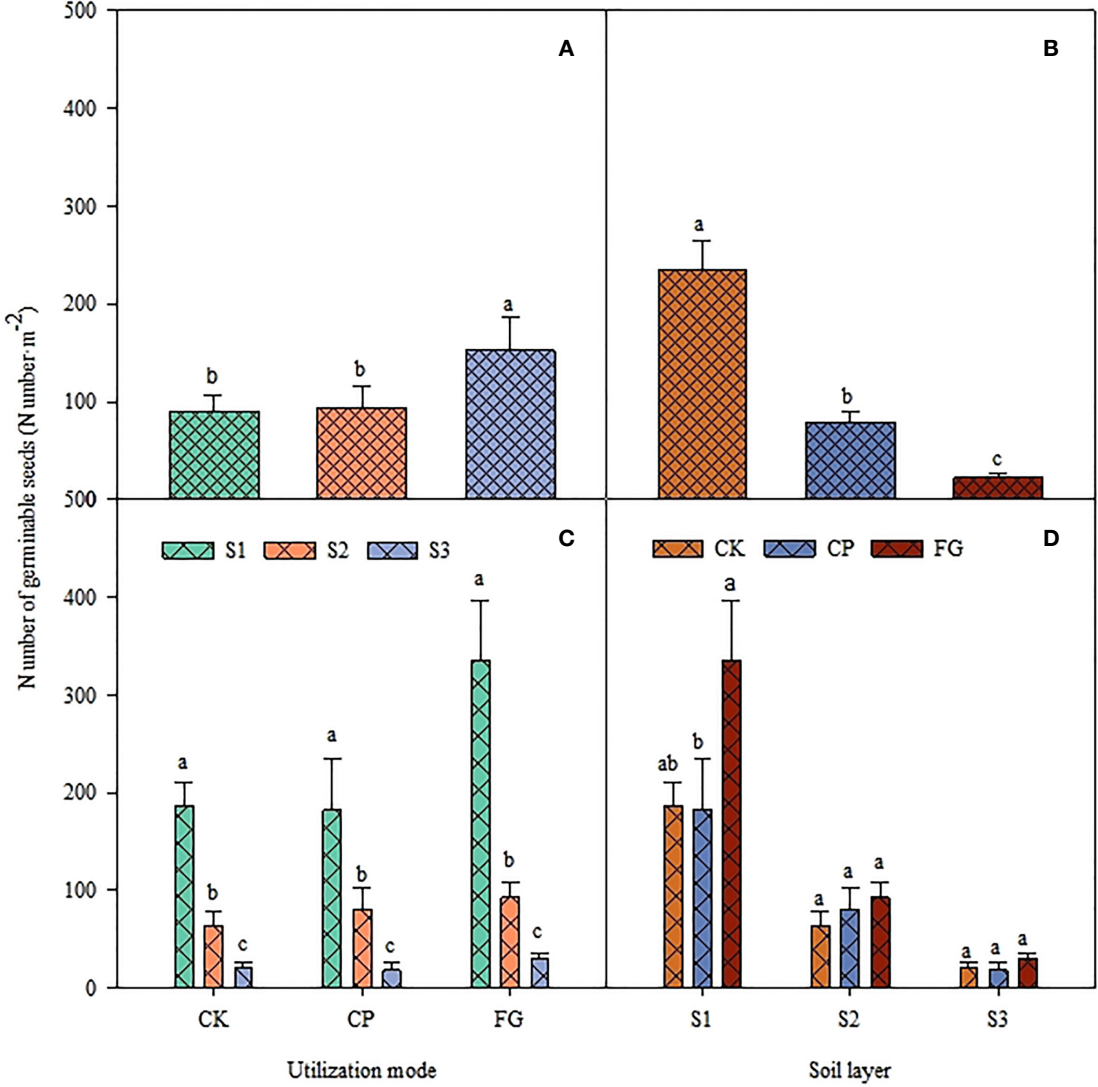
Response of germinable soil seed number of perennial grasses to utilization method and soil depth.

The utilization method and different soil depths significantly affected the germination of perennial grass seeds (**Table 4**, P<0.01). However, the interaction between the two factors did not show a significant impact on perennial grass seed germination (P=0.3899). The number of seed germinations of perennial grasses showed that the trend was CK>CP>FG under different utilization methods, and that of FG was significantly greater than those of CK and CP (**Figure 2-A**). This distribution pattern was obvious at a soil depths of 0-2 cm and not obvious at a soil depths of 2-5 cm (**Figure 2-D**). The distribution pattern of perennial grasses in different soil layers under different utilization methods was still S1>S2>S3, with significant differences (**Figure 2-C**), and the overall perennial grasses in the soil layers also showed significant differences in the order S1>S2>S3. Therefore, it can be seen that free-grazing promoted the germination of the forage soil seed bank of perennial grasses compared with the enclosed grassland, and the germination effect was especially obvious in the 0-2 cm soil layer.

**Table 4:** Analysis of variance (ANOVA) for the effect of utilization method and soil depths on the seed bank of perennial grasses.

Source	DF	SS	MS	F Value	Pr>F	R-Square
Model	8	1633.36	204.17	18.69	<.0001	0.6750
Utilization mode	2	121.94	60.97	5.58	0.0056	
Soil layer	2	1465.74	732.87	67.09	<.0001	
Utilization mode × Soillayer	4	45.68	11.42	1.05	0.3899	
Error	72	786.52	10.92			
Total	80	2419.88				

### 3.4 Effects of different grassland utilization methods on the soil seed bank of the upper growth grasses

**Figure 3:**
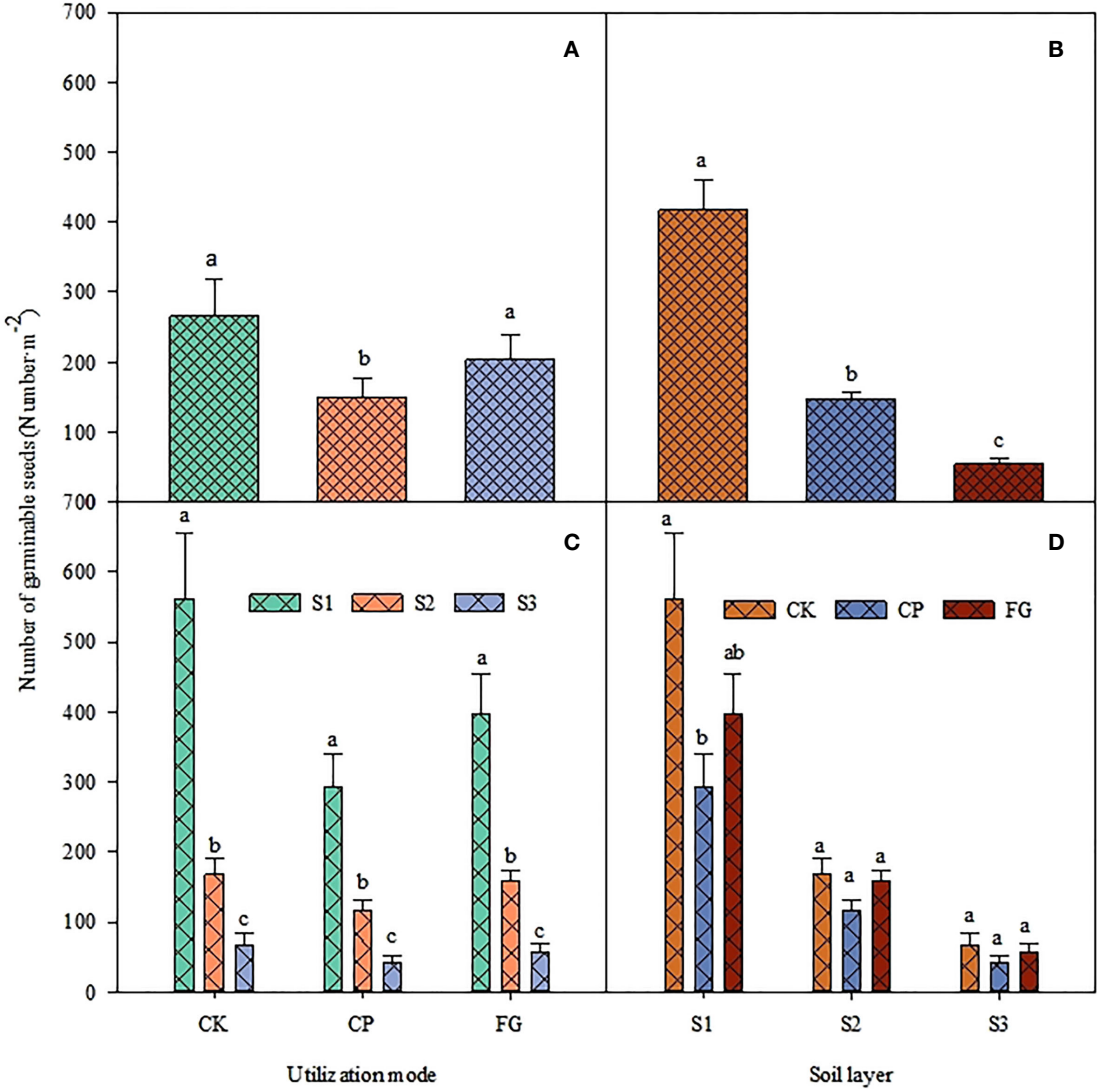
Response of the number of germinable soil seeds of upper growth grasses to utilization method and soil depth.

The effect of utilization method and different soil depths on the seed germination of upper growth grasses forage was highly significant (**Table 5**, P<0.05), and the interaction between the two had no significant effect on the seed germination of upper growth grasses forage. The distribution of the number of seed germinations for different utilization methods for the upper growth grasses was similar to the distribution of the number of germinations in the overall soil seed bank, which was CK>FG>CP, and that of CP was significantly smaller than those of CK and FG, and the difference between CK and FG was not significant (**Figure 3-A**). The distribution also showed a similar distribution pattern at different soil depths; the number of germinations in CK was significantly larger than that in CP in the 0-2 cm soil, and the difference was not significant at other soil depths. Other depths showed the same distribution pattern, but the difference was not significant (**Figure 3-D**). For soil depths, the number of germinable seeds showed the trend 0-2 cm soil > 2-5 cm soil > 5-10 cm soil, the number of germinable seeds in each soil depths differed significantly (**Figure 3-B**), and the distribution pattern for the number of germinable seeds in each soil depths corresponding to different grass utilization methods was the same (**Figure 3-C**). Compared with the enclosed grassland, mowing inhibited the germination of the soil seed bank of the upper multiflora, and the inhibition effect was significant in the 0-2 cm soil layer.

**Table 5:** Analysis of variance (ANOVA) for the effect of utilization method and soil depths on the seed bank of perennial grasses.

Source	DF	SS	MS	F Value	Pr>F	R-Square
Model	8	61.74	7.72	27.83	<.0001	0.7556
Utilization mode	2	3.14	1.57	5.67	0.0052	
Soil layer	2	58.29	29.15	105.09	<.0001	
Utilization mode × Soil layer	4	0.30	0.08	0.27	0.8953	
Error	72	19.97	0.28			
Total	80	81.71				

### 3.5 Interrelationship of different types of germinable soil seed banks

The typical variable of the perennial grass soil seed bank mainly carried information on the soil seed bank in the 5-10 cm soil layer, with a standard loading coefficient of 0.9242, and the typical variable of the soil seed bank for upper growth grasses mainly carried information on the soil seed bank in the 5-10 cm soil layer, with a standard loading coefficient of 0.9584. Since the coefficients of both of the main variables were positive, the correlation between them was positive, and the correlation coefficient was 0.7653, which showed a highly significant correlation (P<0.01). This indicates that there is a consistent linear pattern of variation in the soil seed bank of perennial grasses and upper growth grasses in the 5-10 cm soil layer. Similarly, the influence of soil seed bank of perennial grasses and the upper growth grasses on the soil germinable soil seed bank was also mainly reflected in the 5-10 cm layer, and the degree of correlation reached a highly significant level, with correlation coefficients of 0.7829 and 0.9775, respectively. It can also be seen that there is a strong correlation between the two and the 2-5 cm soil layer of the soil germinable soil seed bank, and the load of the soil germinable soil seed bank in the 2-5 cm soil layer on the whole soil germinable soil seed bank reached − 1.1823, so the effect of perennial grasses and upper growth grasses on the soil germinable seed bank in this soil layer was negatively correlated. The typical correlation between perennial grasses, upper growth grasses, and the soil germinable seed bank extracted 84.58% of the information, which is close to 85% of the statistics and thus has value as a is practical indicator.

## 4 Discussion

### 4.1 Mechanisms by which grassland utilization patterns affect the germinable soil seed bank

In this paper, we first investigated the effects of different grassland utilization methods and different soil depths on the soil seed bank. The results showed that the germination of the soil seed bank was highest in the enclosed grasslands, and grass mowing significantly reduced the germination of the soil seed bank compared to the use of enclosed areas and free-grazing.

The dead matter content in an enclosed grassland is an indicator of grassland ecological management and restoration and is a key factor affecting the seed activity of the soil seed bank (Donath and Eckstein, 2012). The seedling emergence rate and seed retention rate are affected by seed origin and the amount of dead matter present, but the relationship between the presence of dead matter and the renewal of the soil seed bank is complex (Zhang et al., 2017). First, dead material forms a protective layer on the soil surface, regulating the microclimate of the soil surface and changing the moisture and temperature of the soil. (Mackinney, 1929; Sayer, 2006). In contrast, soil temperature and moisture are important factors affecting the longevity of seeds in the soil (Walters, 1998). Related studies on temperature effects have shown that the presence of deadfall reduces soil temperature (Chambers and Macmahon, 1994). An increase in temperature can increase the germination rate of forage seeds to a certain extent (Mackinney, 1929), and within a certain temperature range, the germination rate of plant seeds is positively correlated with temperature (Roberts, 1988). The reason for this is that an increase in temperature may alter the levels of enzymes and hormones in the seeds and thus affect seed germination, and to some extent, an increase in temperature may regulate the levels of hormones such as gibberellin (GA) and abscisic acid (ABA) in plant seeds and thus promote seed germination (Vieira et al., 2017). Abscisic acid inhibits seed germination in soils; while low temperatures cause more abscisic acid to be retained in the seed endosperm, increasing temperatures can eliminate its negative effect on the seed endosperm and thus promote seed germination (Chen et al., 2021). The presence of grassland deadfall can mitigate the effects of a temperature increase on seed germination to a certain extent and can further increase seed survival. In addition to causing changes in soil temperature, the presence of a deadfall layer can also preserve soil moisture (Sayer, 2006); therefore, high soil moisture as well as low temperatures occur beneath the deadfall. At the same moisture level, the water content of seeds increases with decreasing temperature (Roberts and Ellis, 1989). However, practical studies have shown that the survival of different seeds is affected differently by soil temperature and moisture based on the greater degree of influence of these factors on seed longevity and on the effect of litter on plant seed conservation (Mackinney, 1929). Soil moisture content is closely related to fungal growth in soil, with wetter soils promoting fungal growth (Mordecai, 2012), and soil fungi accelerate the rate of seed decay in soils (Wagner and Mitschunas, 2008), but compared with free grazing, enclosed grasslands in meadow steppes may have anoxic conditions due to the saturated soil water content, thereby inhibiting the growth of fungi and increasing the longevity of seeds (Papendick, 1972).

In general, the litter content follows the pattern: enclosed grasslands > grazed grasslands > mowed grasslands. Grazed grasslands typically exhibit sparse litter in the surface soil layer due to the selective consumption by livestock. Grazing leads to shorter grass vegetation and lower vegetation cover, allowing more sunlight penetration. Consequently, grazed grasslands often have lower surface soil moisture content compared to enclosed grasslands. On the other hand, although mowed grasslands may have lower litter content, the absence of grazing allows vegetation to attain greater height and coverage than grazed counterparts. As a result, enclosure promotes improvements in various soil physicochemical properties, water-holding capacity, and soil enzyme activity. This, to a certain extent, elevates grassland quality, consequently enhancing the quantity and density of subterranean seed banks (Hou et al., 2019).

In contrast to fenced-in grasslands, where nutrient reproduction is the mainstay, grazed grasslands are disturbed by livestock, and thus, the plant species in grazed grasslands depend mainly on seed reproduction (Diaz et al., 2004). Plants are inevitably consumed or trampled by livestock in grazing pastures, causing damage to plant reproductive organs or interrupting reproductive processes, which further affects the size of the soil seed bank (Hartnett, 2002). All of the above reasons may cause the number of soil seed banks in grazed grasslands to be lower than that in fenced-in grasslands.

Compared to grazed grasslands, plants in mown grasslands are not frequently disturbed by livestock like in the grazed grasslands. Due to the way the plants reproduce, large numbers of seeds are mown before they can enter the soil, leaving the ecosystem. The amount of seed loss is influenced by the autochthonous shedding rate, which is affected by many factors, such as seed moisture content, seed weight, seed size, and seed morphology (Peart, 1984). In addition to autochthonous causes, mowing height also affects the number of seeds that are removed from the grassland ecosystem, thus reducing the size of the soil soil seed bank.

### 4.2 Effect of grassland utilization patterns on the soil seed bank of perennial grasses

Under the three types of grassland utilization methods, the content of the perennial underground soil seed bank in the free-grazing grassland was significantly higher than that in the first two, and the reason for this may be related to sudden influx of nitrogen. In a study on *Leymus chinensis* meadow grasslands, researchers found that an increase in grassland nitrogen fertilization significantly increased the *Leymus chinensis* seed yield (increased by 79%) and *Leymus chinensis* single seed yield (increased by 40%). An increase in nitrogen can also increase the long-term nitrogen content of grazing-utilized grasslands. Livestock obtain large amounts of nutrients from grazing, and nitrogen is returned to the grassland ecosystem through livestock excreta, so grazing-utilized grasslands have low fertilizer requirements. In contrast, fertilizer application and animal excretion are the main sources of nitrogen in mown grasslands, so in mown grasslands, fertilizer application is generally required to maintain a certain level of productivity. However, nitrogen mineralization is related to the C:N ratio of the soil. The C:N ratio in grasses is relatively high, and grasses in the grass family have much lower mineralization capacities than leguminous grasses. In particular, when a large number of aboveground plant parts are mown, the N cycle in the grassland ecosystem is affected, and a certain degree of N loss occurs. However, the gradual increase in soil organic matter and total N content (heavy grazing causes N loss) that occurs as a result of contributions from plant residues and animal excreta in grazed grassland will to some extent promote the yield of perennial grass seeds and soil seed bank such as *Leymus chinensis*, increasing the proportion of the perennial grass soil seed bank. The presence of litter in fenced grasslands increases the number of perennial forage shoots to promote asexual reproduction of perennial forbs, which in turn increase in dominance in the grassland (Li et al., 2021). The reproductive strategy of perennial grasses in grazed grasslands is dominated by seed reproduction (Diaz et al., 2004); the seed content of perennial grasses in the soil seed bank of grazed grasslands further increases as the seeds are trampled by livestock.

### 4.3 Influence of grassland utilization patterns on the soil seed bank of upper growth grasses

The experimental results revealed that the soil seed bank of the upper growth grasses under the three utilization patterns accounted for a similar proportion of the total soil seed bank (approximately 70%), and the differences under the three utilization patterns were also similar to that of the overall soil seed bank. The upper growth grasses in this experimental area were mainly *Leymus chinensis*, *Artemisia tanacetifolia*, *Astragalus melilotoides*, *Thalictrum squarrosum*, and *Thalictrum squarrosum*. The main reason the patterns were similar is that the seeds are subject to grazing and mechanical harvesting during the physiological process of growth and maturation. The soil seed bank content of the upper growth grasses was more strongly affected by the grass utilization method because the upper growth grasses are generally taller and more susceptible to grazing and mechanical harvesting by grazing livestock.However, from another perspective, the difference between the seed bank of the upper growth grasses under different grassland utilization methods and the overall seed bank had the same trend. This indicates that upper growth grasses have a large impact on the soil seed bank of the grasslands, and a change in the soil seed bank of the upper growth grasses can often determine the overall change in the plant soil seed bank. It is worthwhile to continue to study them in depths.

### 4.4 5Interrelationship of different types of germinable soil seed banks

**Figure 4:**
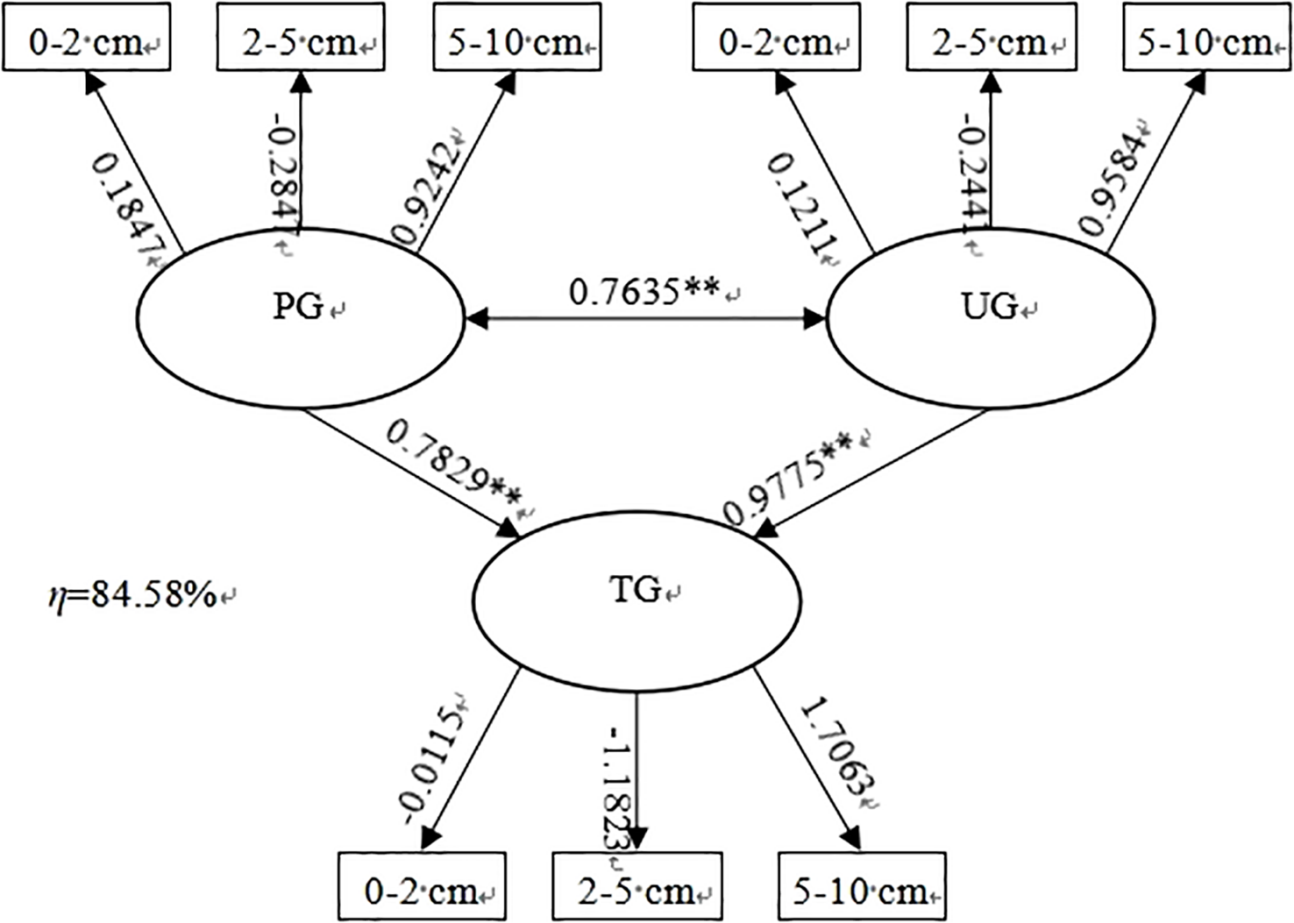
Typical correlation analysis of different components of the soil seed bank.

The soil seed bank of perennial grasses and upper growth grasses had a consistent variation pattern from 5-10 cm, and this variation pattern affected the soil seed bank from 5-10 cm at the same time, indirectly proving that different grassland utilization methods had a strong effect on the soil seed bank in the 0-5 cm soil but a relatively small effect on the soil seed bank below 5 cm, which is more consistent with the results characterized in **Figures 1**-**3**. It was also found that although the load factor of the 2-5 cm soil layer of perennial grass and upper growth grasses was small, the load factor of the 2-5 cm soil seed bank of the soil germinable seed bank was large, and there was a large effect of perennial grass and upper growth grasses on it. Therefore, the perennial grass and upper growth grasses soil seed banks were large but the soil germinable seed bank in the 5-10 cm soil layer was small and was affected by different grassland utilization methods. This implies that the variation in the soil seed bank in the 2-5 cm layer is complex and needs to be further explored in follow-up studies. **Figure 4**

## 5 Conclusions

Different grassland utilization methods and soil depths exerted significant effects on soil seed germination and the germination of perennial grass seeds. The quantity of germinated seeds notably decreased as soil depths increased. Grazing and enclosures led to an increase in soil seed bank density, while mowing resulted in a decrease in soil seed bank density.The number of germinations within the soil seed bank of both the enclosed and free-grazing areas was significantly higher compared to the mown area. Grazing facilitated the germination of the perennial grass soil seed bank, leading to a significant increase in its content. Conversely, mowing hindered the germination of the upper growth grasses soil seed bank.The content of the upper growth grasses soil seed bank, particularly in the 5-10 cm layer, played a substantial role in influencing the overall quantity of soil seed banks to a certain extent. The strong correlations observed among perennial grasses, upper growth grasses, and the soil seed bank germination contributed to an 84.58% information extraction.The outcomes of this study hold significant practical implications for enhancing grassland management practices, safeguarding the ecological environment, and advancing sustainable development.The results of this study hold significant practical implications: Long-term grass cutting practices can lead to a decline in the content of upper growth grasses and perennial grasses in grasslands, resulting in increased instability and decreased grass yield. However, grazing has the potential to enhance the content of perennial grasses, contributing to the stability of grassland quality and yield. This management approach is suitable for the long-term sustainable management of grasslands, offering robust soil protection and environmental benefits.Therefore, adopting a cyclic pattern of grazing and mowing in grassland management may comprehensively enhance the quality and returns of the grass. This management strategy can balance the content of different types of grasses, enhance grassland diversity, and consequently boost the ecosystem functionality of the grassland. This not only aids in sustaining the viability of grassland utilization but also fosters the well-being and sustainable development of the ecological environment.

## Data availability statement

The original contributions presented in the study are included in the article/supplementary material. Further inquiries can be directed to the corresponding authors.

## Author contributions

RY: Conceptualization, Writing - Review & Editing, Funding acquisition. TY: Formal analysis, Writing - Original Draft. HL: Conceptualization, Validation. SL: Formal analysis, Resources, Visualization, Data Curation. BC: Resources. YW: Project administration. GQ: Formal analysis, Data Curation. ZW: Supervision, Methodology. LX: Resources. XZ: Project administration. GY: Project administration. XX: Validation, Writing - Review & Editing, Funding acquisition. All authors contributed to the article and approved the submitted version.
